# Retention of service users on opioid substitution therapy in the City of Tshwane, South Africa

**DOI:** 10.4102/phcfm.v15i1.3392

**Published:** 2023-01-30

**Authors:** Daniela S. Goeieman, Dimakatso S. Nonyane, Doudou K. Nzaumvila, Michelle N. S. Janse van Rensburg

**Affiliations:** 1 Department of Family Medicine, School of Medicine, Faculty of Health Sciences, University of Pretoria, Tshwane, South Africa

**Keywords:** Opioid dependence, opioid substitution therapy (OST), retention, harm reduction, Community-Oriented Primary Care (COPC), Community-Oriented Substance Use Programme (COSUP), methadone, nyaope

## Abstract

**Background:**

Opioid substitution therapy (OST) is evidence-based treatment for opioid use disorders and, when taken as maintenance therapy, has proven health and social benefits. The benefits of OST are achieved through the retention of service users in the treatment programme.

**Aim:**

To identify factors that affected retention of service users who had OST interrupted in less than 6 months of being in an OST programme.

**Setting:**

This qualitative study was conducted with 19 service users from eight Community-Oriented Substance Use Programme (COSUP) sites in the City of Tshwane, Gauteng, South Africa.

**Methods:**

Participants were COSUP service users who had interrupted OST in less than 6 months since initiation and were purposefully selected from all COSUP sites. Demographic information was obtained and four focus group discussions covered challenges of OST retention. Discussions were recorded, transcribed and qualitatively analysed using Attride-Stirling’s thematic networks framework.

**Results:**

The 19 participants were all male, mostly black African, with a mean age of 26 years. Facilitators of retention in OST were individual readiness to change OST accessibility, positive family and peer support, treatment monitoring, understanding and managing expectations of service users, contribution in society and meaningful opportunities for engagement. Barriers were the cost of OST, bureaucracy within the programme, inability to communicate challenges timeously and effectively to treatment providers, boredom, cravings and poverty.

**Conclusion:**

Opioid substitution therapy programmes can ensure a holistic approach to prevent and treat harms related to illicit opioid use if they remain person-centred and are well-funded.

**Contribution:**

Understanding the barriers to, and facilitators of retention on OST can contribute to improved community-based service delivery.

## Introduction

Globally, there are about 11.3 million people who inject drugs (PWID), and opioid use causes two-thirds of drug-related deaths.^1^ The trafficking of heroin in Africa has increased since 2013, with South Africa being one of the main transit countries for consignments destined for North America and Europe via air.^1^ The South African Community Epidemiology Network on Drug Use releases reports biannually containing data from treatment centres across South Africa.^2^ In their second report of 2019, opiates (particularly heroin) were the leading substance of use for treatment enrolment in Gauteng at 36%, service users were predominantly black African (74%), with a mean of age 26 years, and only 14% of the total population that accessed services were female.^3^ ‘Nyaope’ is the street name for a cocktail of drugs containing heroin, used in the City of Tshwane, and it is relatively cheap and easily accessible.

Inpatient rehabilitation may be considered an option for the treatment of substance use disorders, but there are insufficient drug rehabilitation services in the public sector in South Africa, leading to long waiting periods for admission and private services being unaffordable for the majority.^1^ In addition, opioid-use disorders have disappointing outcomes, such as high return-to-use rates, when inpatient rehabilitation and detoxification methods are used as a one-size-fits-all approach to treatment.^4,5,6^

Institutions such as Harm Reduction International have called for global change in the field of addiction care as far back as the Eighties. They describe harm reduction as ‘policies, programmes and practices that aim primarily to reduce the adverse health, social and economic consequences of the use of legal and illegal psychoactive drugs without necessarily reducing drug consumption’.^7^ The focus is not on the substance use, but on positive change in a nonjudgemental environment that does not require abstinence as a precursor for support.^7^

This holistic view draws parallels with Norman Zinberg’s multisystemic theoretical framework of *drug, set, and setting*. Equal consideration and importance is given to the drug, the mindset (set) and the environment (setting) when assessing and providing services to substance users.^8^ The strength of and how the drug is used; the individual’s genetic disposition, experiences and behaviour (psychological disorders, child abuse, intention of the person having the experience, etc.); and the social environment (social class, peer culture or physical environment) all play significant roles in the drug experience and path to substance use disorder.^9,10^

Whilst substance use can be because of or lead to unfavourable life circumstances and challenging socio-economic realities (e.g. unemployment, poverty, homelessness, etc.), it is its ability to drive diseases of poverty (most importantly tuberculosis [TB], human immunodeficiency virus [HIV], acquired immunodeficiency syndrome [AIDS] and malnutrition) that concerns health stakeholders.^1,11,12,13,14,15^ Although data are limited, the number of PWID in South Africa is over 75 000, and this key population accounts for 1.3% of new HIV infections.^14^ Of the three major metros in South Africa, Tshwane has a 21% HIV prevalence and the highest hepatitis C prevalence amongst PWID, who were found to be predominantly black men living on the city streets.^11^ Efforts are being made to move towards a pro–harm reduction policy, as reflected in national policies, including the Drug Master Plan of 2019–2024, the mention of opioid substitution therapy (OST) for maintenance in the Department of Health’s TB/HIV Strategic Plan 2017–2022, and the South African Hospital Level Standard Treatment Guidelines and Essential Medicines List for Adults.^12,13,14,15^

Opioid substitution therapy, as a component of the harm-reduction approach, is an evidence-based intervention for managing opioid dependence. Opioid substitution therapy as maintenance therapy is provided by skilled treatment providers in regulated outpatient clinics or in community-based settings.^1,7,16^ Clinicians support service users to receive either an opioid oral agonist (e.g. methadone) or a partial agonist (e.g. buprenorphine, buprenorphine–naloxone).^16^ This maintenance treatment approach is more effective than OST for detoxification, withdrawal or opioid antagonist treatment in reducing illicit opioid use, and it improves retention in treatment programmes.^17^ Opioid substitution therapy decreases HIV and viral hepatitis C incidence, improves TB and HIV treatment adherence rates, reduces high-risk practices and improves people’s quality of life.^6,11,13,18^

Western and South East Asian countries have, over the past 40 years, developed research, resources, experience and the political will to diversify treatment options with the addition of OST at the national level amid the opioid crisis.^13,17,18^ In the South African context, national implementation has not yet been reached, although the National Department of Health has drafted an OST plan that includes roll-out. Until implemented, this leaves a privileged few accessing OST through their own financial means or community-based initiatives, such as the Community-Oriented Substance Use Programme (COSUP).^11,19^

The Community-Oriented Substance Use Programme was established in 2016 in response to the City of Tshwane’s need to address the growing opioid crisis.^20^ The programme is funded by the City and implemented by the University of Pretoria’s Family Medicine Department as part of the Community-Oriented Primary Care (COPC) Research Unit. The Community-Oriented Substance Use Programme offers an alternative to formal inpatient, abstinence-based rehabilitation for opioid use. The programme uses the COPC approach to provide primary healthcare, OST for maintenance and psychosocial services (e.g. counselling and skills development), with the aim of reducing harms caused by opioid use.^20^

Through an interdisciplinary team approach, COSUP has managed to introduce OST at the primary healthcare level with the support of health and social institutions in the public, nongovernment, academic and private sectors across the City of Tshwane. This has allowed for integrated interventions targeted at service users and task shifting to ensure access to skilled and knowledgeable professionals and team members. One of the priority areas for COSUP has been the development of a sustainable plan to roll out maintenance OST at the primary care level.

A service user is prescribed an OST dose by the clinician. Treatment starts on a low dose (5 mg – 10 mg), which is then up-titrated to reach the therapeutic effect and an adequate maintenance dose.^20^ The criteria needed for service users to qualify for OST funding at the time of the study were attendance for three weeks (approximately six treatment sessions) and reduction in the quantity of illicit opioids smoked or injected to prepare for the OST initiation dose of 20 mg – 40 mg for methadone and 2 mg – 8 mg for buprenorphine-naloxone. A COSUP study conducted from December 2016 to September 2018, looking only at service users on methadone, found that 61.6% of the study participants were not retained after six months.^21^ Access to funded methadone facilitated retention, and a dose greater than 50 mg improved retention.^21^

While it is clear from the study that COSUP had experienced problems similar to other OST programmes in the country – for example, human resource constraints, a lack of funds to supply service users with doses that will subdue the use of illicit opioids and challenges in adequately managing comorbidities – there was a need to find clarity on what the service users themselves perceive to be factors affecting their retention.^19,20^ For better service delivery and retention, there needs to be an understanding of what personal- or project-related factors contributed to service users not being retained on OST in COSUP, hence the motivation to engage service users in discussions around OST and produce this qualitative study. The aim of the study was to explore the response to services by opioid-dependent service users who received OST, so as to understand facilitators of and barriers to retention whilst on OST from the participants’ perspectives.

## Research method and design

### Study design

This qualitative research was a descriptive, exploratory study designed to gather information from COSUP service users who were on OST about factors that influenced retention.

### Setting

Seventeen COSUP sites exist across the City of Tshwane in low- to middle-income areas. Each of the community-based sites provides harm-reduction services, including OST, and the team consists of clinical associates, primary health care nurses and doctors (family physicians and registrars) to manage OST and medical conditions; social workers (including students and auxiliaries) to assist with family and community reintegration; and peer educators and community health workers to facilitate community education and destigmatise substance use. At some sites, occupational therapists and occupational therapy students address contextual aspects, such as the constructive use of time and vocational skills development; psychologists and interns have been placed for behavioural therapy; and psychiatrists assess and manage dual diagnosis patients.

All 17 COSUP sites were requested to participate in the research, but only eight responded. These were situated in peri-urban areas (Eersterust, Mamelodi, Atteridgeville, Soshanguve, Olievenhoutbosch and Ga-Rankuwa), with sites operating from community settings, including community health centres, a regional hospital and nongovernment organisation (NGO) sites. The other two sites were located in inner city settings, including a homeless shelter (Hatfield) and community clinic (Sunnyside). All the sites form part of the metropolitan area of Tshwane, situated in the South African province of Gauteng.

### Study population

Research participants were residents from around Tshwane and were attending at their closest COSUP site.

### Sampling

Purposeful sampling^22^ was used, and inclusion criteria for this study were service users who had OST (methadone or buprenorphine–naloxone) interrupted in less than 6 months, with or without re-initiation of OST. Site managers were requested by the researchers to contact all the eligible service users based on available OST retention data and explain the aim and objectives of the study. Nineteen participants from the eight sites were willing to participate. They were given a date to avail themselves for the focus group discussions (FGDs) at a neutral venue, and COSUP transport was provided from their respective COSUP sites to the venue and back.

### Data collection

#### Focus group procedures

Four FGDs were conducted to determine the challenges faced by COSUP service users on the OST programme, specifically the history behind their OST being interrupted in less than six months of initiation.

The FGDs were conducted at the University of Pretoria’s Mamelodi campus and Kalafong Hospital Regional Training Centre. To ensure a safe and free setting, the four FGDs were conducted by a senior researcher in the COPC Research Unit, who, although working in COSUP, did not have prior engagement with the participants. The senior researcher was experienced in running FGDs and was assisted by a clinical associate from one of the COSUP sites for the three FGDs that did not include participants from her site. To ensure that participants from the clinical associate’s site did not feel restricted in providing honest answers, a COSUP social worker from a different site with no participants assisted for that particular FGD.

Open-ended questions for the interview guide were prepared using Zinberg’s tripartite model^8,23^ (*drug, set,* and *setting*). The questions posed pertained to what the participants thought the factors were that affected their retention in the OST programme. Where more detail was required, the facilitators probed further. Participants were encouraged to express themselves in the language that they felt most comfortable in, as the facilitators complemented each other by being fluent in the languages spoken (English, Sepedi, Setswana, isiZulu and Afrikaans). The FGDs each lasted for about 2 h. Focus groups were audio-recorded with the permission of the participants.

#### Demographic data

In addition, written informed consent was obtained to access the participant-administered questionnaire that is used for service user enrolment into the COSUP programme to collect demographic information of the participants (race, biological sex, age, education level, type of housing, sources of income, route of taking opioids, other substances used and type of OST).

### Data analysis

The FGD recordings were transcribed verbatim and translated, where necessary, by a research assistant (master’s degree student) not involved in COSUP. The recording of one of the FGDs was faulty, but thorough notes had been taken by the facilitators, so information from that FGD was retained. The transcribed text and researcher notes were scrutinised for salient information that was then coded by one of the authors who did not conduct the FGDs. These codes were then further analysed and reflected upon by the researchers who had conducted the FGDs for triangulation. Similar codes were grouped together in order for themes to emerge. A thematic analysis was conducted using Attride-Stirling’s thematic networks framework.^24^ This technique used web-like illustrations to systematically explore, summarise and interpret the main themes.^24^ The first draft of the article was sent to another researcher in COSUP, who is an expert in substance use harm reduction, who critically engaged with the analysis and findings to enhance trustworthiness.

### Ethical considerations

This study falls under a larger study about the development, application and implementation of COSUP services, and ethical approval was obtained from the Research Ethics Committee of the Faculty of Health Science at the University of Pretoria (reference number 83/2017). The FGD facilitators explained the purpose of the focus groups and details of the study before obtaining written informed consent from all participants and before starting the discussion. To ensure just access, all participants were assisted with transport to the two sites where FGDs took place by the COPC Research Unit driver, fetching participants from their various COSUP sites and taking them back to their sites after the FGD sessions. Participants were not compensated financially for their participation.

## Findings

### Demographics

The 19 COSUP clients who participated in the four focus groups had similar demographics (see Table 1) compared to the majority of service users accessing services from COSUP, that is, male with the average age of 26 years and exiting the school system at Grade 10. The largest racial grouping were black Africans (*n* = 16). All mentioned their family homes as primary residence, but some were homeless or living in shelters at the time. Thirteen participants chose to smoke *nyaope*, with five choosing to inject the drug. Only one participant had formal employment at that time, working as a cleaner.

**TABLE 1:** Participant demographic information.

Participant characteristics	*N* = 19
Race	black people = 16
	Mixed race people = 2
	white people = 1
	Indian people = 0
	Other = 0
Biological sex	Male = 19
	Female = 0
Age†	15–25 years = 2
	26–35 years = 12
	36–45 years = 4
	46–55 years = 0
	> 55 years = 0
Level of education†	Primary school
	Grade 6 = 1
	High school
	Grade 9 = 1
	Grade 10–11 = 10
	Grade 12 = 5
	Diploma = 1
	Undergraduate = 0
	Postgraduate = 0
Housing	Brick house = 14
	Informal housing = 1
	Homeless or homeless shelter = 4
Sources of income	Informal work = 0
	Employed = 1
	Unemployed = 16
	Social or disability grant = 2
Route used to take opioids†	Smoking = 13
	Injecting = 5
Other substances whilst on OST	Cannabis = 1
	Tobacco = 18
Type of OST	Methadone = 17
	Buprenorphine–naloxone = 2

OST, opioid substitution therapy.

† , Data missing for one participant.

### Themes

**FIGURE 1:**
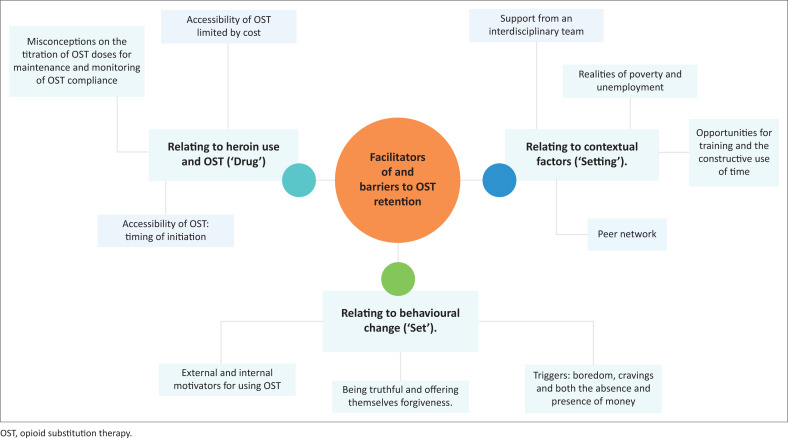
Thematic network for facilitators of and barriers to opioid substitution therapy retention.

The following themes were interpreted from discussions with the participants and helped to develop an understanding of the facilitators and barriers that affected their retention on OST (Figure 1 illustrates the thematic network).

#### Theme 1: Retention of opioid substitution therapy for maintenance is affected by the accessibility of opioid substitution therapy, the monitoring of treatment and the understanding and expectations of the service users regarding opioid substitution therapy (‘drug’)

**Accessibility limited by cost:** The majority of the participants were on methadone funded by COSUP, whilst a few, prior to their return to using illicit opioids, were purchasing their own methadone or were on buprenorphine–naloxone funded by the programme. All participants in this study agreed that the main barrier to access and stay on OST (in the absence of sponsorship by COSUP) was the price, with the cost of a 500 mL bottle of methadone being over R500 per month at the time of the research. As one participant stated:

‘COSUP is doing a very big job [*sic*] because some of us cannot afford the medications.’ (P1, Male FGD1)

Without the financial assistance from COSUP, participants would have had great difficulty staying on the programme, successfully overcoming opioid use disorder and preventing the associated harms. One participant explained:

‘I got stress[*ed*], I had to include my mom but I [*didn’t*] know what plan we [*were*] going to make. Maybe they will request money from my uncle or request her boss to lend her money, but plan come out somewhere [*sic*]. I remember I made two hundred rand from the street and asked my mom for three hundred and she gave [*it to*] me. So, I had to attend for six months and I only struggle[*d*] for the first time, so I still had to pay for the next five months. But then I talked to them about the challenge of raising the remaining months’ funds. Then they told me that there [*is*] some funding coming. They will consider people like me first who are committed to the programme, but can’t afford medication on their own. That’s when my stress level was reduced, because after explaining that money challenge to my mom, she was also stressed and then coming back with the news of the assistance, she was happy.’ (P2, Male, FGD1)

**Accessibility limited by timing:** At the time, the criteria needed for OST funding required attendance for three weeks (approximately six treatment sessions) and reduction in the number of *nyaope* bags smoked or injected to prepare for the OST initiation dose. The waiting period and initiation phase was a high-risk period for treatment cessation:

‘I had a problem, because before coming to COSUP for [*those*] three weeks … I could not come before I smoke[*d*], so I had to get the fix and when you wake up you had to run around looking for cash to buy so I can smoke [*sic*].’ (P2, Male, FGD1)

Participants attributed a strong willpower and motivation for staying the course:

‘[…*T*]hen they asked me if I am serious, then after that they took me back to the programme and started from the first three weeks.’ (P3, Male, FGD1)

‘[…*I*]t’s not three weeks but only six sessions. But it leads to three weeks because others come maybe twice in a week; that is the reason it leads to three weeks. But if they see the commitment, they quickly start medication on you [*sic*] even before the six sessions.’ (P4, Male, FGD1)

**Misconceptions on the titration of opioid substitution therapy doses for maintenance and monitoring of opioid substitution therapy compliance:** For participants who wanted to abstain from using illicit opioids, the titration of the OST dose to therapeutic effect proved to be challenging, which caused service users to substitute with *nyaope*:

‘[…*I*]ssue with [*a*] small dosage when they start, it is not good because the medication, it takes time to work, unlike when you smoke. So if they could start us with [*a*] good dosage, which allow[*s*] us to meet the demand of our smoke[*ing*], [*it*] will lead to us not double use and relapse [*sic*].’ (P5, Male, FGD2)

Although participants achieved the desired functioning, for example, improved self-care whilst on OST, some said that they still felt the need to be intoxicated:

‘There was a time where I realised that medication had no problem, but the problem was us, because there was a time where I took my medication and I would have the thought that I am not high because I haven’t smoke[*d*] and went to look for smoke [*sic*], but after I started taking 20 mLs [*40 mg*], those psychological craving move [*sic*] away.’ (P5, Male, FGD2)

Alternatively, service users reported a fear of developing an ‘addiction to methadone’, which contributed to reluctance to increased doses. Participants whose therapeutic dose was inadequately monitored by the clinician admitted to developing a pattern of using both OST and *nyaope* over time. This was also true for those whose take-home dose was not supervised. A participant elaborated about his dilemma:

‘I relapse because … they don’t give you the chance of reducing the dosage they give you … You drink the medicine and they don’t check if you are still using or not. And you get used to smoking and using medication to a point that when you smoke, you want methadone after. Until I decided to leave methadone, because I could not use both methadone and *nyaope* because the [*withdrawal*] craving was getting too high. Because I drink 30 mLs here and go to smoke maybe five packets. December [*sic*] I said I don’t want these things and I started taking methadone until now, but once in a while I do take one packet, but I don’t smoke regularly.’ (P6, Male, FGD2)

Service users’ initial understanding of what will be expected of them by the COSUP team played a role in the freedom of service users to be open and honest. Despite the health education offered on the harms, participants either continued this pattern of use, chose to follow OST correctly as advised or discontinued OST for *nyaope*:

‘So when I take my drugs, I can get high enough because I am used to taking both methadone and *nyaope*, so I have to take both in order for me to be high enough. So I had a friend who was addicted to both heroin and methadone, so when he smoke[*d*] heroin he was not high enough, so he use[*d*] to take them both until [*they*] refuse[*d*] to give him the methadone.’ (P7, Male, FGD2)

#### Theme 2: Achieving the required behavioural change rests on the readiness to change, along with a strong internal or external motivation for change (‘set’)

**Internal and external motivators for using opioid substitution therapy:** For some participants, the motivation to change at the onset was initially external, for example, pressure from family, especially the matriarch:

‘[…*T*]he parent collects the medication on behalf of the child, but you find that the child does not use it when they are at home.’ (P8, Male, FGD2)

‘I was doing it for my mother, as she is the one who took me to COSUP. So in the morning, while she is around, I would take methadone, but later on when she went to work I would go to my friends and we would smoke.’ (P5, Male, FGD2)

Yet, for others, they had specific personal reasons to pursue change:

‘I do it for myself and do wish that one day I can get a job and take care of my mom, because, if I am not mistaken, next year she will be starting with her social grant and I see she is [*too*] old to work and support me.’ (P3, Male, FGD1)

**Being truthful and offering themselves forgiveness:** The participants stressed the importance of being truthful. Dishonesty and the inability to communicate about their challenges led to participants not being managed appropriately, returning to use and the therapeutic relationship being strained. The following exchange between the focus group facilitator and participants illustrates this:

P7, Male, FGD2: ‘For me as a user we need to be truthful, because we are not honest when we are registering. The truth is that we are the problem, because we get shy to speak the truth and that leads us into getting the wrong dosage.’

FGD facilitator: ‘What made you move from not being honest to being honest now, what changed?’

P7, Male, FGD2: ‘… now we realised that for us to lie, we also get the wrong medication or dosage [*sic*].’

FGD facilitator: ‘what makes you get to the point that *“now I must speak the truth?”.*’

P7, Male, FGD2: ‘It started when I was arrested after I robbed someone [*of their*] money so that I [*could*] get a pack to smoke. So from the time I was in the waiting cell, I told myself I had to be straight and after I was released I spoke the truth and now [*I*] am here.’

P8, Male, FGD2: ‘For me it was after realising the damage I was doing to [*my*] parents and the community I was staying with.’

FGD facilitator: ‘Do you have a specific moment that led you to change?’

P8, Male, FGD2: ‘Yes, I do. I had to change because I realised that I am failing even to sit with family and talk. Then I realised I need to change … to have the chance to be with my family. And after that, I realised a change in myself and the people around me. Now they even send me to buy things, while previously they never did that and now [*I*] am able even [*sic*] to help my siblings with their school work.’

**Triggers for using nyaope:** Participants reported that they struggled with cravings and boredom, and they acknowledged their struggles in managing their money. Participants showed insight regarding cravings:

‘We need to understand as users that, as we are taking the medication, we will have a struggle and craving to use.’ (P6, Male, FGD2)

‘[…*A*] big thing is the mindset; if the mindset is still with the heroin you will still go back to heroin …’ (P9, Male, FGD3)

The risk of use of *nyaope* was increased by boredom:

‘Sitting at home is dangerous and it leads to many relapse[*s*].’ (P11, Male, FGD3)

Participants acknowledged the habit of using their money on drugs:

‘[…*W*]hen I get money I go back to smoking.’ (P10, Male, FGD3)

‘[…*N*]ot used to going to the movies, you are already in that mindset that, oh R50.00 you see two bags, so we need that mentor [*to*] motivate us on what or how to use our extra cash.’ (P10, Male, FGD3)

Participants felt that they used *nyaope* as a result of lack of willpower against these triggers, but they also mentioned environmental barriers as challenges, which leads to the third theme.

#### Theme 3: Environmental factors and supportive networks (‘setting’)

Service users’ substance use goals, be they abstinence, using less or mitigating harm and risk, were reported to be impacted by their conditioning through different encounters in an unsupportive or positively enabling environment. Poor outcomes were said to be a result of drug use in friendship circles, lack of recreational opportunities, food insecurity and unemployment.

**Peer network:** These participants’ responses bring to the fore how people on OST live in community with other people, some of whom may use drugs:

‘I went back, because my problem is that I quit using, but I still hang around with the same people at the same spot that I used to hang around while I was using because they are my friend[*s*].’ (P3, Male, FGD1)

‘I was still in the same environment with them and when you go out, you meet them on the street and [*they*] request R5 from you and, you see, they take you to a spot to buy and smoke, and tomorrow the same thing …’ (P12, Male, FGD3)

‘Those who relapse, for me, is that they start taking medication, but they don’t have the change of scenery and they don’t want to put in the necessary effort, because without that you will always be tempted to try, but if you change the friends and hang out spot, then you can change and quit drugs, but it require[*s*] your own effort in all of that.’ (P7, Male, FGD2)

**Realities of poverty and unemployment:** Not being able to sustain oneself makes adherence to treatment difficult:

‘[*M*]ethadone does give you stomach cramps because of hunger and so it is confused as stomach cramps from heroin. So they want to top up, many people think it is heroin and run back to it while it was just hunger, so if only they had something to eat … methadone does not work because people are not eating.’ (P9, Male, FGD3)

The lack of opportunities for meaningful engagement, particularly in the constructive use of time and opportunities for income generation, were difficult for participants:

‘[…*W*]e just don’t want to sit at home and do nothing … at least something to keep us busy ….’ (P10, Male, FGD3)

Contextual factors identified for good treatment outcomes were reconciling with family, positive peer relationships, support within COSUP, the ability to contribute in society, the availability of life-skill’s training (e.g. from within COSUP and from community, NGO and academic partners), and opportunities for engagement that brings meaning to the individual (e.g. constructive use of time, learning, income-generation or self-enterprise).

**Support from an interdisciplinary team:** The importance of bio-psychosocial support and life skills development was emphasised by participants:

‘[…*G*]o to social workers or COSUP to find encouragement if I fail to find advice from the family members.’ (P7, Male, FGD2)

‘[…*A*]ttend OT [*occupational therapy*] classes. We do life skills programme[*s*], stress management, how to be assertive, jobs searching skills and [*the OT*] suggest books to read.’ (P2, Male, FGD1)

The unique and essential contribution of peer educators was also highlighted:

‘[…*S*]omeone who was once a user talking to you about the process and encouraging such [*a*] person to go through the process, it becomes easy, unlike talking to someone who has never walk[*ed*] through the smoking route.’ (P8, Male, FGD2)

‘[…*F*]ormer users as peer educators and participants in the group sessions.’ (P5, Male, FGD2)

**Opportunities for training and the constructive use of time:** Engagement in activities that brought meaning to participants and provided opportunities for personal development was valued:

‘[…*D*]uring life skills we have a building where we help[*ed*] fixing [*sic*] [*it*] and we clean[*ed*] [*it*] out oursel[*ves*] and it really help[*ed*] a lot … help on that project, it gave us a sense of belonging and it really help[*ed*] us. It was, like, fun and we were coming together for something and paint[*ing*], clean[*ing*] and do[*ing*] the gardening and it keeps us busy. I think those areas need to be protected and every time we pass by the area we feel proud that we are the ones who did that garden or painting.’ (P9, Male, FGD3)

‘[…*S*]omething to keep us busy like [*training*], where you go to school for eight hours. And it’s like not sitting at home where you are sitting and doing nothing. At least you are doing something, not like when you are on the street.’ (P6, Male, FGD2)

‘What helped me … I used to be busy with the computers [sic], fixing and building computers.’ (P9, Male, FGD3)

## Discussion

This is the first qualitative study looking at OST retention in a community-oriented harm reduction programme across the City of Tshwane. Much like other South African research produced on harm reduction and OST, the participants were predominantly African men, under 35 years of age, single and unemployed.^11,19,21^ These similarities are not to say that those who do not fit this particular demographic are not at risk. Interestingly, however, according to a recent quantitative study regarding OST retention in Tshwane, the white population group and those living in the inner city (as opposed to peri-urban settings) had better odds of retention.^21^

The themes that emerged from this research pertained to the service users’ relationships with *nyaope* and OST (*drug*) whilst in COSUP; service users’ personality traits, expectations, and readiness for change (*set*); and the environmental factors relevant to service users (*setting*). These are similar to themes found in the experiences and retention of service users in other OST community programmes.^25^ (see Figure 1).

### ‘Drug’

Participants experienced challenges regarding the accessibility of OST, particularly in terms of cost. Without the financial assistance from COSUP to access OST, the treatment was found to be unaffordable, with participants relying heavily on COSUP-funded OST to stay on treatment. Possibly because of programmatic cost limitations, clinicians were administering a maintenance dose that was lower than the recommended dose of between 60 mg and 120 mg of methadone syrup or 8 mg of suboxone (buprenorphine and naloxone).^15^ Government funds are the most important financing method for services to address substance use disorder in South Africa, and state OST funding is still mostly limited to inpatient detoxification.^26^ It would seem that cost has a negative effect on most service users because of the low doses of OST prescribed, and the need for and heavy reliance on COSUP-funded OST for treatment.

Challenges were also experienced in the timing of initiation, as well as dosing of OST, particularly at the stabilisation phase, where the ideal OST dose is still being determined to avoid intoxication and withdrawal and manage the risk of overdose. Despite what participants deemed to be a trusting therapeutic relationship with the COSUP team, it seemed as if it did not always extend to clinical decision-making, as participants found it difficult to alert the team of inadequate doses of OST, causing cravings and breakthrough withdrawals that led them to ‘topping up’ with *nyaope*. From service users’ perspectives, the likelihood of concurrent use of methadone and *nyaope* was attributed to deficiency in close monitoring and early intervention by clinicians in addressing the use of OST and ‘topping-up’ with *nyaope* to get intoxicated; inadequate supervision of take-home doses; and self-medicating to cope with homelessness, food insecurity and life stressors. These factors have been reported in other studies to contribute to returning to using *nyaope* and misuse of OST.^21,27^

It is to be expected that some OST service users will abstain from illicit opioids or use illicit opioids infrequently, providing that they receive high quality OST services (i.e. appropriate dose with appropriate support).^2,16,25^ Service users need to play an active role in their treatment, as it is key in how they perceive the OST.^25^ The World Health Organization reiterates that showing a service user respect, providing them with knowledge and working through issues in a systematic fashion are enhanced by including service users in clinical decision-making to reach an agreement that will secure adherence to treatment.^17^ The necessary reassurance and health education from clinicians, social workers and peers in COSUP is crucial.^21^

### ‘Set’

When considering service users’ mindsets and behavioural changes, it is important to note that some participants understand a lack of retention to be a lack of willpower. The perception of service providers of topping-up or using *nyaope* whilst on OST as ‘bad’ or ‘failing’ contributes to what could be interpreted as negative pressure. Success in treatment does not rest purely on being strong-willed but on a service user’s ability to develop strategies to control their environment to preserve their willpower.^28^ Great effort and commitment are required in respectfully addressing maladaptive thoughts and behaviours, because thoughts of shame, regret and fear of alienation made participants hide their use of illicit drugs from clinicians and family. These thoughts are counter-productive to the goal of OST and the harm reduction approach, which is improving health outcomes and meeting service users at whatever stage of change without judgement.^7^

### ‘Setting’

Once service users’ self-esteem and strategies improved, they could be adherent to treatment, take the necessary initiative to seek opportunities to be industrious and develop a sense of purpose. Participants felt that pragmatic support, such as prevocational (time-management) and vocational training (e.g. financial planning), would have assisted them to make informed and empowered decisions once they started earning money from finding employment or their own income-generation efforts. Once a service user enters a process of recovery with strategies and enthusiasm, the sustainability of this process requires an environment that prioritises skills development and affords training opportunities at strategic times for each individual.^28^

South Africa is faced with the devastating reality of high levels of poverty and youth unemployment, which makes the goal of self-sustainability increasingly difficult^29^ for a homeless, substance-dependent South African. Illicit substance users’ ability to be self-reliant is negatively influenced by an unfavourable environment. If the lack of shelter, finances, food security and support for illicit substance users is not addressed and remedied, it could render them without viable options around their use of either illicit opioids or OST.^20^ Engagement in meaningful activities, such as vocational and life skills training, and the constructive use of time, such as group and sport activities, provide opportunities for growth and support and contribute to health and well-being whilst dealing with the realities of poverty.^30^

The participants’ input regarding OST and the other factors that affect their ability to stay on treatment is crucial information for COSUP, and for treatment providers in Africa, where this is still a novel treatment.^19,31^

### Recommendations

This article makes the following recommendations:

- Opioid substitution therapy services should consider three particular areas affecting retention: access to and appropriate monitoring of OST (neither under- nor over-monitoring); the individual’s motivation and readiness to change along with support in doing so; and how much their environment influences their outcomes in treatment.
- Extensive education about OST and substance use to service providers, service users, family and community should be prioritised. Opioid substitution therapy as part of harm reduction services needs to be holistic and integrated to be effective.
- Treatment providers need to prioritise and continuously monitor access to OST. Service users need shorter waiting periods for treatment initiation, and bureaucracy should not affect the ability for clinicians to get a service user on treatment. Opioid substitution therapy treatment should continue for as long as the patient benefits from treatment, wishes to continue and suffers no adverse reactions.
- Opioid substitution therapy should be available to anyone with an opioid use disorder, with a focus initially on high-burden areas. Implementing this goal at the national health budget level should allow for programmes such as COSUP to offer harm reduction services in primary care settings and use community pharmacy dispensing opportunities to increase the number of opioid-dependent people who access OST when needed.
- The value of therapeutic group interventions as a means for facilitating behavioural change within a community-based harm reduction programme such as COSUP should be further investigated.
- The sustained implementation of programmes to better develop skills for and facilitate engagement in meaningful activities and income-generation opportunities for COSUP service users should be prioritised and researched further.

### Strengths and limitations of the study

The participants were free to express themselves in their home language and participation did not affect their access to COSUP services. Unfortunately, there were no female representation in the FGDs to interrogate the assumption of disproportionate access and type of treatment services. In addition, service users who were retained on OST were not interviewed to further assess factors that enhanced retention. Whilst more empirical data are needed to understand the barriers and facilitators for OST retention in South Africa, especially in other contexts and with a variety of service users (e.g. in terms of age and sex), this study can contribute to responsive community-based OST programmes that meet the needs of South African service users or those in similar contexts.

## Conclusions

This qualitative study explored the perceived facilitators and barriers of retention amongst service users accessing OST at a primary healthcare level. These were considered in terms of the drug itself (the service user’s relationship with *nyaope* and the accessibility of OST whilst in COSUP), the service users’ own behaviours and readiness for change (e.g. motivation, trust and triggers for use of *nyaope*), and the environmental factors relevant to service users that either hindered (e.g. the realities of poverty) or supported their retention to OST (e.g. a supportive interdisciplinary team and opportunities for meaningful engagement).

Opioid substitution therapy programmes can be used to ensure a more holistic approach to prevent and treat harms related to illicit opioid use if they remain person-centred and responsive and are well-funded.
