# Corrigendum: Retention of service users on opioid substitution therapy in the City of Tshwane, South Africa

**DOI:** 10.4102/phcfm.v15i1.4033

**Published:** 2023-11-30

**Authors:** Daniela S. Goeieman, Dimakatso S. Nonyane, Doudou K. Nzaumvila, Michelle N.S. Janse van Rensburg

**Affiliations:** 1 Department of Family Medicine, School of Medicine, Faculty of Health Sciences, University of Pretoria, Tshwane, South Africa; 2 Department of Family Medicine, Faculty of Health Sciences, University of the Witwatersrand, Johannesburg, South Africa; 3 Department of Family Medicine and Primary Health Care, Sefako Makgatho Health Sciences University, Pretoria, South Africa

In the published article Goeieman DS, Nonyane DS, Nzaumvila DK, Van Rensburg MNS. Retention of service users on opioid substitution therapy in the City of Tshwane, South Africa. Afr J Prim Health Care Fam Med. 2023;15(1), a3392. https://doi.org/10.4102/phcfm.v15i1.3392, there was an error regarding the affiliations for Daniela S. Goeieman and Doudou K. Nzaumvila.

Instead of:

**Authors:**

Daniela S. Goeieman^1^ https://orcid.org/0000-0002-8647-1350

Dimakatso S. Nonyane^1^ https://orcid.org/0000-0001-6702-6326

Doudou K. Nzaumvila^1^ https://orcid.org/0000-0002-3257-0329

Michelle N.S. Janse van Rensburg^1^ https://orcid.org/0000-0002-0371-1022

**Affiliations:**

^1^Department of Family Medicine, School of Medicine, Faculty of Health Sciences, University of Pretoria, Tshwane, South Africa

It should be:

**Authors:**

Daniela S. Goeieman^1,2^ https://orcid.org/0000-0002-8647-1350

Dimakatso S. Nonyane^1^ https://orcid.org/0000-0001-6702-6326

Doudou K. Nzaumvila^3^ https://orcid.org/0000-0002-3257-0329

Michelle N.S. Janse van Rensburg^1^ https://orcid.org/0000-0002-0371-1022

**Affiliations**:

^1^Department of Family Medicine, School of Medicine, Faculty of Health Sciences, University of Pretoria, Tshwane, South Africa

^2^Department of Family Medicine, Faculty of Health Sciences, University of the Witwatersrand, Johannesburg, South Africa

^3^Department of Family Medicine and Primary Health Care, Sefako Makgatho Health Sciences University, Pretoria, South Africa

The authors apologise for this error. The correction does not change the study’s findings of significance or overall interpretation of the study’s results or the scientific conclusions of the article in any way.

